# Two steps for scoring a point: Creating and converting opportunities in invasion team sports

**DOI:** 10.1371/journal.pone.0240419

**Published:** 2020-10-15

**Authors:** Leonardo Lamas, José Vitor Senatore, Gilbert Fellingham

**Affiliations:** 1 Faculty of Physical Education, University of Brasilia, Brasilia, Distrito Federal, Brazil; 2 Department of Statistics, Brigham Young University, Provo, Utah, United States of America; Technical University of Madrid, SPAIN

## Abstract

In invasion team sports, scoring efficiency depends on the ability to create scoring opportunities and to convert them into goals or points. Scoring performance varies across sports and comparisons among them are little. In this paper we compare creation and conversion of scoring opportunities in different team sports and genders. Box-score data from six sports [basketball, handball, water polo, field hockey, football, ice hockey] (328 teams, 5723 games, both genders) were standardized by “per ball possession”. We applied Bayesian methods to compute the posterior distributions of shots per possession (SHTpPOS), points per shot (PTSpSHT) and points per possession (PTSpPOS). We evaluated differences for these three variables among sports, between genders and the correlation between every pair of them. Inter-sports evaluation evidenced basketball, handball, ice hockey and water polo are sports with a high probability of creating shots (SHTpPOS—p(robability) > 0.65). Complementary, ice hockey, field hockey and football are sports with a low probability of converting shots (PTSpSHT—0.05 < p < 0.26). Despite the distinct results among sports for creating and converting opportunities, all sports in both genders, converged to a scoring efficiency (PTSpPOS) below 0.5. In the genders’ comparison, men are more efficient in creating opportunities than women, except in water polo. For scoring efficiency, differences between men and women are fewer. Results prevent generalization about differences in scoring efficiency between genders. Finally, creation and conversion have low correlation in sports with high shot creation probabilities (basketball and ice hockey). In these sports, scoring is not limited by the number of shots taken but rather by their accuracy. For sports with low shot creation probabilities (soccer and men field hockey), creation and conversion presented higher correlation. Evidences contribute to increase coaches’ understanding about scoring tactics’ challenges in team sports and design practices accordingly.

## Introduction

In invasion team sports, scoring is the positive outcome of creating an opportunity to score and using that opportunity to covert a goal/point [1, 2]. The frequency with which teams create and convert opportunities in different team sports is influenced by the number of players, size and type of the game field, scoring zone and other features imposed by the rules of the game.

For instance, in association football, the frequency of scoring opportunities is lower than in others sports such as basketball. Thus, increasing scoring opportunities is critical in football, fomenting extensive debate over offensive strategies [3–7]. In contrast, in basketball, many more scoring opportunities are created than are available in football, but their conversion into points through improvements in the quality of opportunities created is the primary challenge [8, 9]. Ice hockey is a sport in which the frequency of scoring opportunities and conversion of those opportunities is quite disparate [10, 11]. Although perceptions over offensive efficiency of team sports quite often pervade the common-sense debate, comparative assessment of scoring performance seems to lack scientific reasoning, leading to superficial understanding of the diversity of the creation-conversion paradigm in different team sports.

Some support for studying scoring patterns may be found in the rapid growth of research about tactics in each team sport individually [12]. Scoring efficiency issues are frequently addressed, e.g. in basketball [8, 9], handball [13, 14], waterpolo [15–17], field hockey [18], ice hockey [10] and association football [5–7, 19, 20]. Particularly, some studies have accounted for the time-related context of the opposition and evidenced its impact in the match dynamics and the scoring patterns coordination [21–24]. In handball and basketball, the game-scoring coordination of opposing teams seems to increase as the game unfolds from first to second half [23, 24]. In volleyball, it was observed a higher scoring efficiency in serves during the game’s last 15 rallies for higher level teams [22]. In football, the relative phase of ball possessions shifted from anti-phase, in the initial drawing moment of the game, to in-phase, in the winning/losing situation at the last term of the game. Winning teams also distinguished their passing and shot effectiveness from the drawing moment before a comeback to other periods [21].

The evolving understanding about scoring efficiency variables and their respective influential factors whithin each team sport contrasts with the relative paucity of comparative analysis across team sports [25]. Standardized comparisons among team sports may contribute to precisely establish the differences in their performances and should be of particular interest for sports with similarities in their playing structures [26], such as focused-target invasion team sports (e.g. basketball, handball, football association, water polo) or open-ended target invasion team sports (e.g. rugby, American Football) [26–28]. Merritt and Clauset [25] assessed American sports’ leagues (American football, basketball and ice hockey) and demonstrated common patterns for the frequency and balance of points scored despite the highly distinct playing structure of the sports. Nonetheless, how scoring opportunities are created and converted into points across sports are not addressed.

Still, there are few studies focused on women [29–31] or encompassing both genders [32, 33] in the analysis. Gender differences in team sports performance may have a multi-factorial explanation [33], influenced by physical [34], hormonal [35], psychological [36] among other factors. In net sports, previous studies demonstrated that these differences may lead to distinct adjustments in men’s and women’s strategies and tactics [34], impacting the game dynamics [30, 37] and the type of scoring actions performed [38]. Despite that, both genders may present similar efficiency [39]. In invasion team sports, differences between men’s and women’s likelihood of scoring related variables remains unclear.

Recalling the common-sense debate, invasion team sports differ considerably in creating and converting efficiencies (e.g. association football and ice hockey). Thus, scoring outcomes should be interpreted in terms of both of these components, in order to provide insights for further performance improvements [2]. Hence, the goal of this study was to compare scoring efficiencies, encompassing both creating and converting opportunities, among invasion team sports and between genders within the same sport.

## Materials and methods

We analyzed ball possession efficiency by assessing creation and conversion of scoring opportunities in six different team sports: i) basketball; ii) handball; iii) water polo; iv) field hockey; v) association football; and vi) ice hockey. For basketball, handball, water polo and field hockey public data sets of official competitions were available for both men and women, allowing for within sport gender comparisons.

In contrast to the other sports considered, basketball scores are weighted and can earn one, two or three points. For this reason, we analyzed basketball scoring in two different ways—with weighted points and unweighted points (every score having a value of one). In the rest of this document, references to basketball are for weighted scores. Basketball_unweighted_ will always be used when the analysis is of unweighted basketball data.

### Possession

The frequency of ball possessions was estimated from box score data following procedures used in basketball [40, 41], with adjustments for each sport. Despite small variations in the official definition in each sport, a possession starts when one team gains control (or possession) of the ball and ends when that team gives up control of the ball [40] through a score attempt or losing possession via an error, a violation or a foul. Normalizing via possessions mitigates the influence of game pace [26, 42], which may considerably vary among sports and even within a sport [6, 43]. We also calculated the rates of possessions per minute among sports to inform about their matches’ dynamics.

Estimated ball possessions were used to standardize comparisons among sports based on the frequency of all ending of possession actions available in box-scores of each sport. Generally, ball possessions terminate in one of two types of ending actions—score attempts or turnovers. For these ending events, sports present specific actions. Specifically in basketball, only one of up to three free throws attempts will be a possession ending action. Thus, we applied a correction factor of 0.44, an estimated value indicating the percentage of free throws that terminate a possession [40, 41]. We now present the assumed complete set of end of possession actions for each sport and the respective calculation of the number of ball possessions.

- Basketball ending actions: i) shots (field goal attempt, free throw attempt); ii) turnovers (opponent steal, bad pass, ball handling error, violation, offensive foul).
  $${\text{Basketball}}_{\text{bp}}=\;\text{field}\;\text{goal}\;\text{attempts}\;+\;(0.44*\text{free}\;\text{throw}\;\text{attempt})\;+\;\text{turnovers}.$$
  Or, alternatively,
  $${\text{Basketball}}_{\text{unweighted}}{}_{{}_{\text{bp}}}=\text{field}\;\text{goal}\;\text{attempts}+\text{free}\;\text{throw}\;\text{attempt}+\text{turnovers}.$$
- Handball ending actions: i) shots (goals, saves, off-target, on-post, blocked); ii) turnovers (dispossessed, bad pass, ball handling error, violation, offensive foul)
  $${\text{Handball}}_{\text{bp}}=\text{shots}+\text{turnovers}$$
- Water polo ending actions: i) shots (goals, saves, off-target, on-post, blocked); ii)turnovers (dispossessed, inaccurate pass, violation).
  $${\text{Water}\;\text{polo}}_{\text{bp}}=\text{shots}+\text{turnovers}$$
- Field hockey ending actions: i) shots (goals, saves, off-target, on post, blocked); ii) turnovers (opponent tackle, bad pass, violation).
  $${\text{Field}\;\text{hockey}}_{\text{bp}}=\text{shots}+\text{turnovers}$$
- Association football ending actions: i) shots (goals, saves, off-target, on post, blocked); ii) turnovers (inaccurate passes, dispossessed, bad control, off-sides);
  $${\text{Association}\;\text{football}}_{\text{bp}}=\text{shots}+\text{turnovers}$$
- Ice hockey ending actions: i) shots (goals, saves, missed shots, blocked shots); ii) turnovers (dispossessed, inaccurate passes, bad control, violations); iii) fouls (physical fouls, restraining fouls, stick fouls, game flow fouls, other fouls).
  $${\text{Ice}\;\text{hockey}}_{\text{bp}}=\text{shots}+\text{turnovers}$$

### Scoring efficiency

We defined three variables related to scoring efficiency: i) shots per possession (SHTpPOS); ii) points per shot (PTSpSHT); iii) points per possession (PTSpPOS). SHTpPOS measures the efficiency of creating scoring opportunities. PTSpSHT measures the efficiency of converting shots into scores. Finally, PTSpPOS measures the overall scoring efficiency (creating and converting scoring opportunities) in a ball possession.

### Sample

Archive data were obtained from open-access official webpages from the International and National Federations of each sport. We compiled and assessed box-score data for six sports. Contest frequency for each sport was as follows: i) women’s basketball: 2019 American WNBA regular season (12 teams; 408 games); ii) men’s basketball: 2018-2019 American NBA regular season (32 teams; 2,460 games); iii) women’s handball: 2017-2019 Russian Handball Super League (22 teams; 292 games); iv) men’s handball: 2018-2019 German Bundesliga (18 teams; 306 games); v) women’s water polo: 2013, 2015, 2017 and 2019 Women’s World Championships (80 teams; 236 games); vi) men’s water polo: 2013, 2015, 2017 and 2019 Men’s World Championships (80 teams; 236 games); vii) women’s field hockey: 2019 Women’s Pro League (9 teams; 67 games); viii) men’s field hockey: 2019 Men’s Pro League and 2018 Men’s World Cup (24 teams, 75 games); ix) men’s association football: 2018-2019 English Premier League (20 teams; 380 games); x) men’s ice hockey: 2018-2019 NHL regular season (31 teams; 1,263 games). In the case where data from national championships were not available, we used data from national teams during world championships. If more than one world championship was used, a national team in two different world championships was considered to be two different teams in the sample, since players and coaches were not the same. See S1 File for details.

### Data analysis

We applied Bayesian methods to compute the posterior distributions of the means and correlations of the variables of interest, for each sport and gender. The use of the Bayesian paradigm seems especially appropriate when evaluating observational data. Bayesian methods allow the researcher to update probability densities in a coherent way based on current data. Thus, we are not constrained to define a null hypothesis, but rather we just examine what we believe the current probability structure to be based on our observations. Probabilities of differences between means and correlations were computed using differences of the Markov Chain Monte Carlo (MCMC) draws computed for the parameters. For every comparison, we used the term ‘significantly different’ when the posterior probability of the difference exceeding 0 was > 0.9. We used the computer program JAGS [44] to compute chains drawn from the posterior distributions of interest. Plots were made using the computer program R [45].

For each sport and gender, four variables were computed: (1) possessions per minute, (2) shots per possession, (3) points per shot, and (4) points per possession. Thus, we used a multivariate likelihood to compute the appropriate posterior distributions.

First, we transformed all the data points into standard normal variates by subtracting the sample means and dividing by the sample standard deviations. Then the likelihood for the transformed variables was as follows:

$$\begin{array}{c} y_{1:4_{i}}∼MultivariateNormal\left(\mu_{1:4},\Sigma_{4\times 4}\right) \end{array}$$ (1)

The priors were as follows:

$$\begin{array}{c} \mu_{1:4}∼Normal\left(0,1\right)\\ \Sigma^{-1}∼Wishart\left(R,4\right) \end{array}$$

where

$$R^{-1}=\;[\begin{array}{cccc} 1 & .2 & .2 & .2\\ .2 & 1 & .2 & .2\\ .2 & .2 & 1 & .2\\ .2 & .2 & .2 & 1 \end{array}]$$

To compute posterior chains for the variable means, the chains were back transformed into the original metric. Posterior chains for the correlations did not need to be back transformed. In all the text that follows, if we present a single number summary, it is the mean of the posterior distribution of the variable under discussion.

## Results

Scoring efficiency is first contextualized in terms of the possession exchange rates in each sport. Possession exchanges were normalized per minute, to avoid the influence of different game durations among sports. Then, we present the comparative results of the posterior probabilities associated with the three scoring efficiency variables. Next, we compare the efficiencies between genders based on their respective posterior distributions. Finally, for each sport and gender, we display the correlations between the variables for creating opportunities (SHTpPOS), converting opportunities (PTSpSHT) and overall possession efficiency (PTspPOS).

### Ball possessions

**Fig 1:**
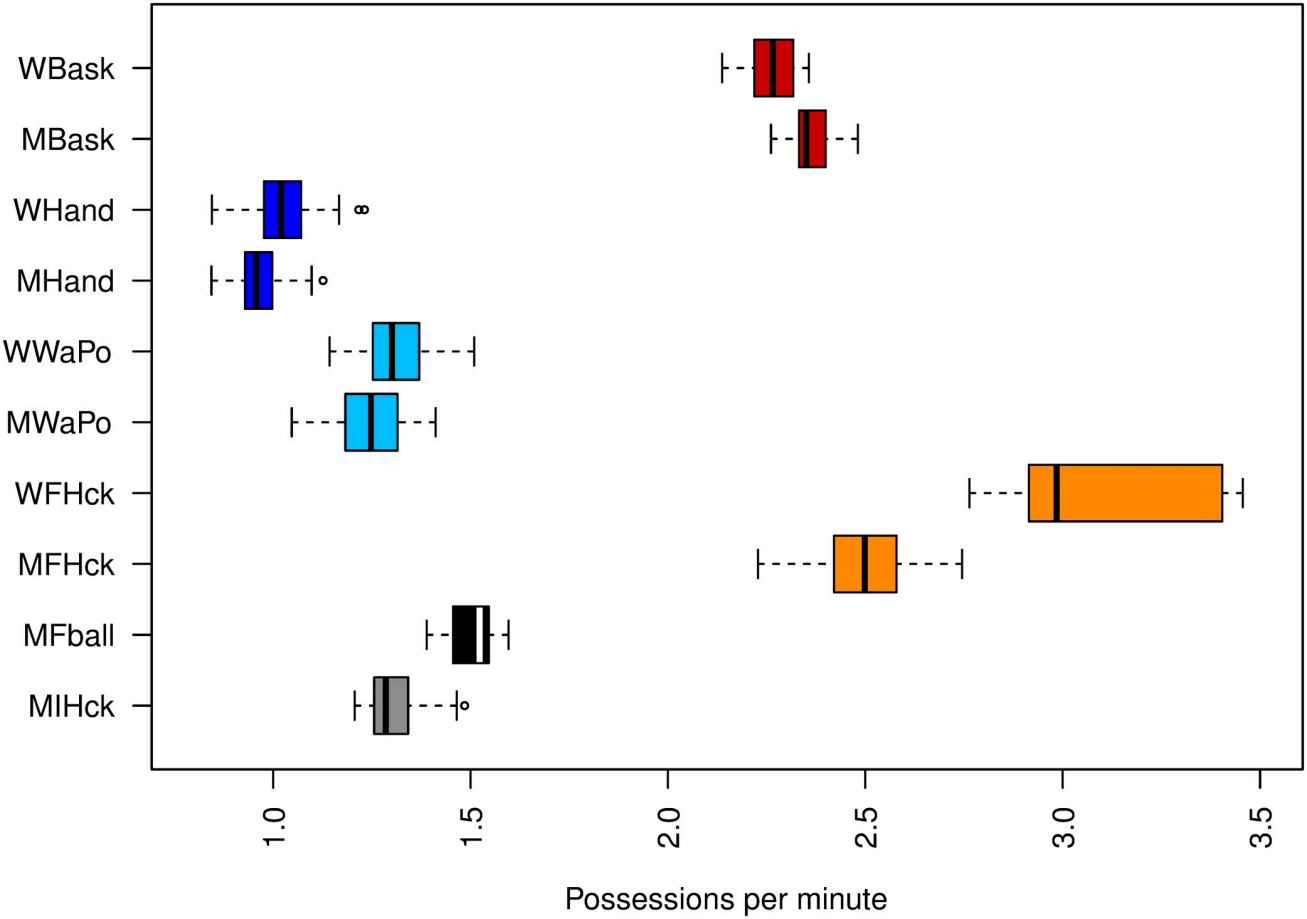
Boxplots of ball possessions per minute in each sport.

Fig 1 displays boxplots of the data relative to ball possessions for each team and sport on a per minute basis. Women’s field hockey had the greatest frequency of possession exchanges per minute (average: 3.10 possessions per minute). Men’s field hockey had the second greatest exchange rate (2.49), although significantly lower than women field hockey (p > 0.9). Basketball also had high exchange rates, with men (2.36) having a significantly higher exchange rate than women (2.26), (p > 0.9). Football (1.50), ice hockey (1.31), women water polo (1.31) and male water polo (1.25) all had ball possessions between 1.25-1.50 per minute. The lowest possession rates were found for handball (women: 1.02, men: 0.96), with women having a significantly higher rate than men (p > 0.9).

### Efficiency: Comparison among sports

#### Shots per possession

**Fig 2:**
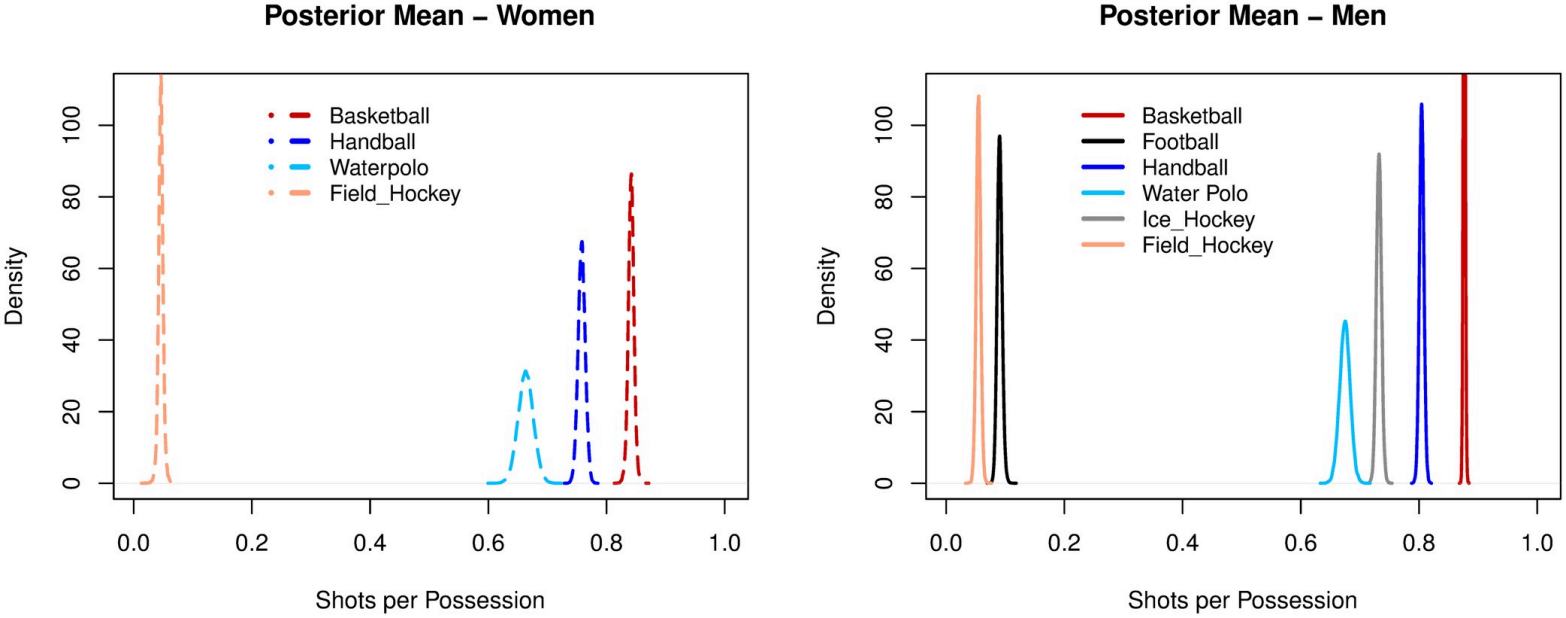
Posterior densities of the mean parameter of shots per possession among sports.

Basketball (women: 0.84, men: 0.87), handball (women: 0.76, men: 0.80), water polo (women: 0.66, men: 0.67) and ice hockey (men: 0.73) had mean frequencies higher than 0.60 for SHTpPOS, both for women and men. Field hockey (women: 0.04, men: 0.05) and association football (0.09) had mean frequencies below 0.10. The ranking of sports for SHTpPOS efficiency was the same for both genders (i.e. basketball—the highest ratio—to field hockey, the lowest), see Fig 2.

#### Points per shot

**Fig 3:**
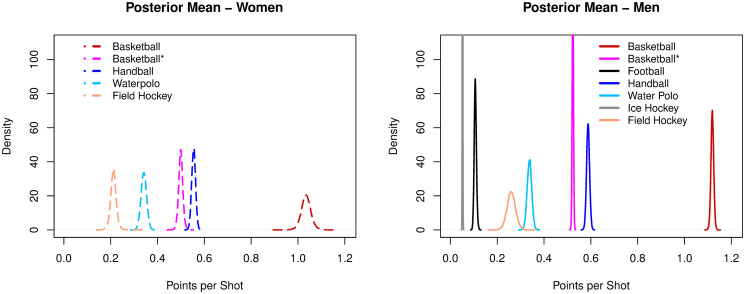
Posterior densities of the mean parameter of points per shot among sports.

Basketball had the highest ratios of PTSpSHT, both for women (1.03) and men (1.11), when considering actual points scored. If each successful shot only counted one point, then women and men ratios dropped, respectively, to 0.49 and 0.52. Basketball unweighted ratios were significantly below handball (p > 0.9) (women: 0.55, men: 0.58). Field hockey had ratios just above 0.2 (women: 0.21; men: 0.26), followed by football (0.11) and ice hockey (0.05), with the lowest values of points per shot, see Fig 3.

#### Points per possession

**Fig 4:**
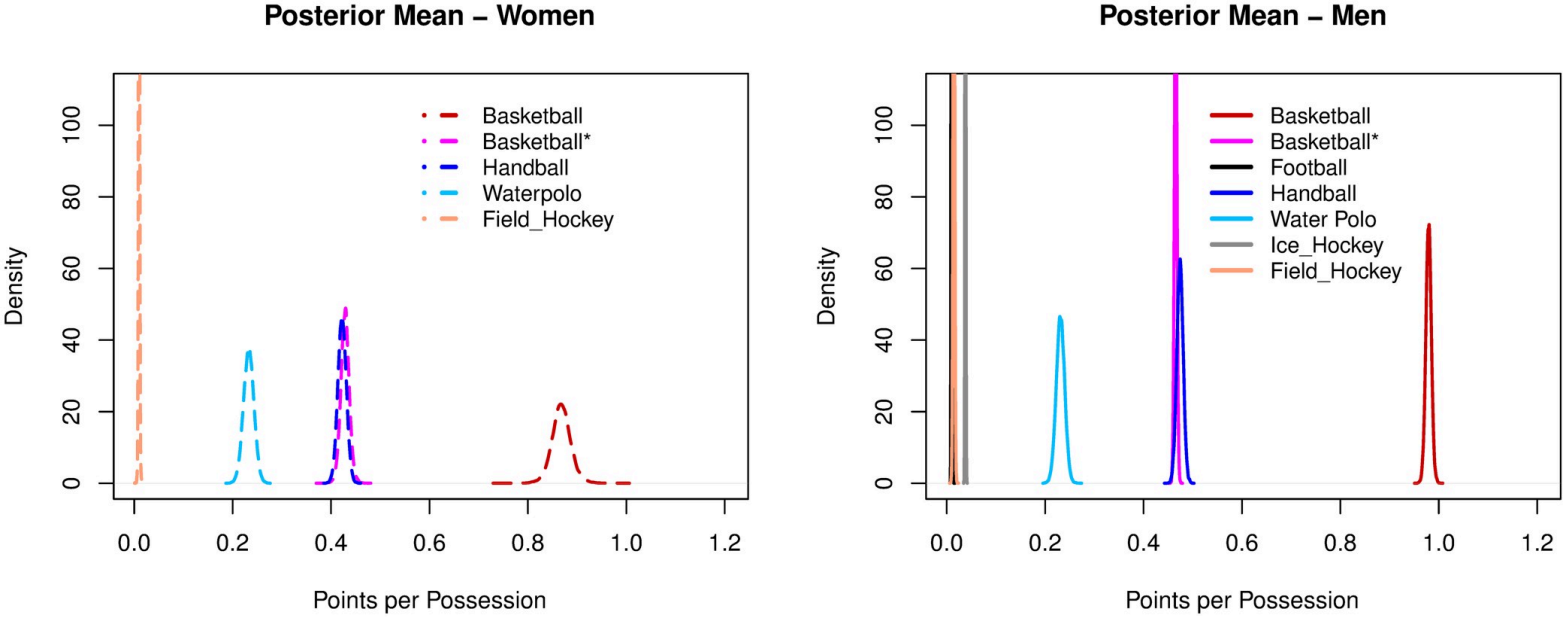
Posterior densities of the mean parameter of points per possession among sports.

Basketball unweighted (women: 0.43; men: 0.46) and handball (women: 0.42; men: 0.47) had similar ratios of points per possession. Water polo was just greater than 0.2 (women: 0.23; men: 0.23). The lowest ratios of PTSpPOS were observed for field hockey (women: 0.01; men: 0.01), football (men: 0.01) and ice hockey (men: 0.03). Basketball with weighted scores was the only sport to exceed 0.5 points per possession (women: 0.87, men: 0.98), see Fig 4.

### Efficiency: Comparison between genders

#### Shots per possession

**Fig 5:**
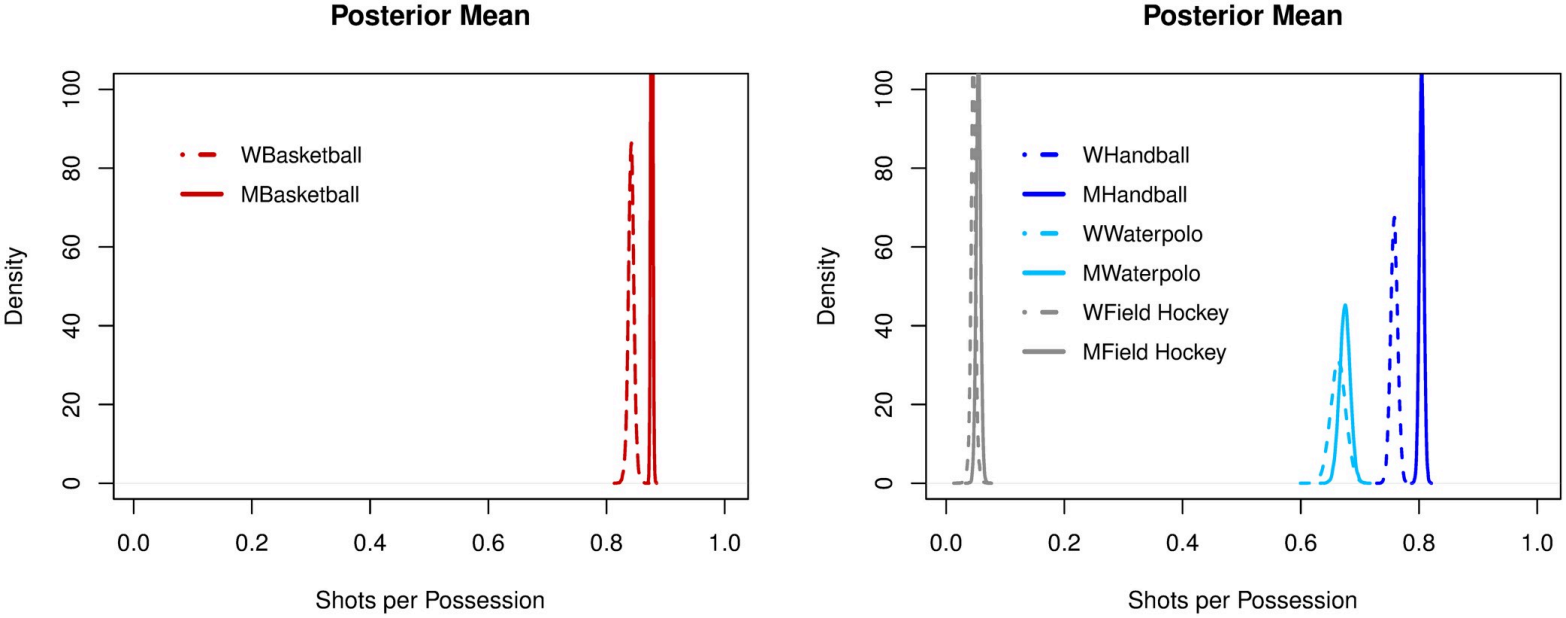
Posterior densities of the mean parameter of shots per possession across genders.

In both basketball and handball, men had significantly higher rates of SHTpPOS than women (p > 0.9). In water polo, women and men had similar rates. In field hockey, rates were both low but significantly higher for men (p > 0.9), see Fig 5.

#### Points per shot

**Fig 6:**
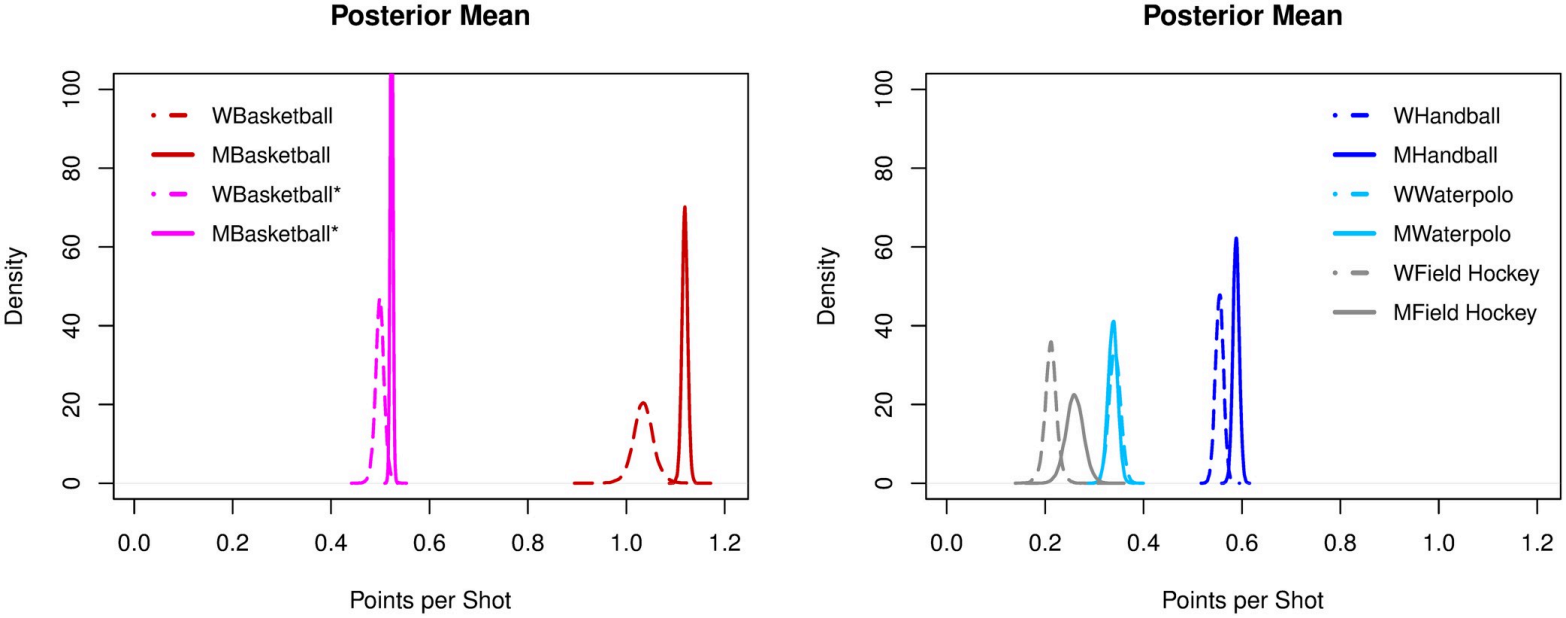
Posterior densities of the mean parameter of points per shot across genders.

For PTSpSHT, basketball, handball and field hockey all had significantly higher rates for men than for women (p > 0.9). Basketball (unweighted) and water polo had similar rates for both genders, see Fig 6.

#### Points per possession

**Fig 7:**
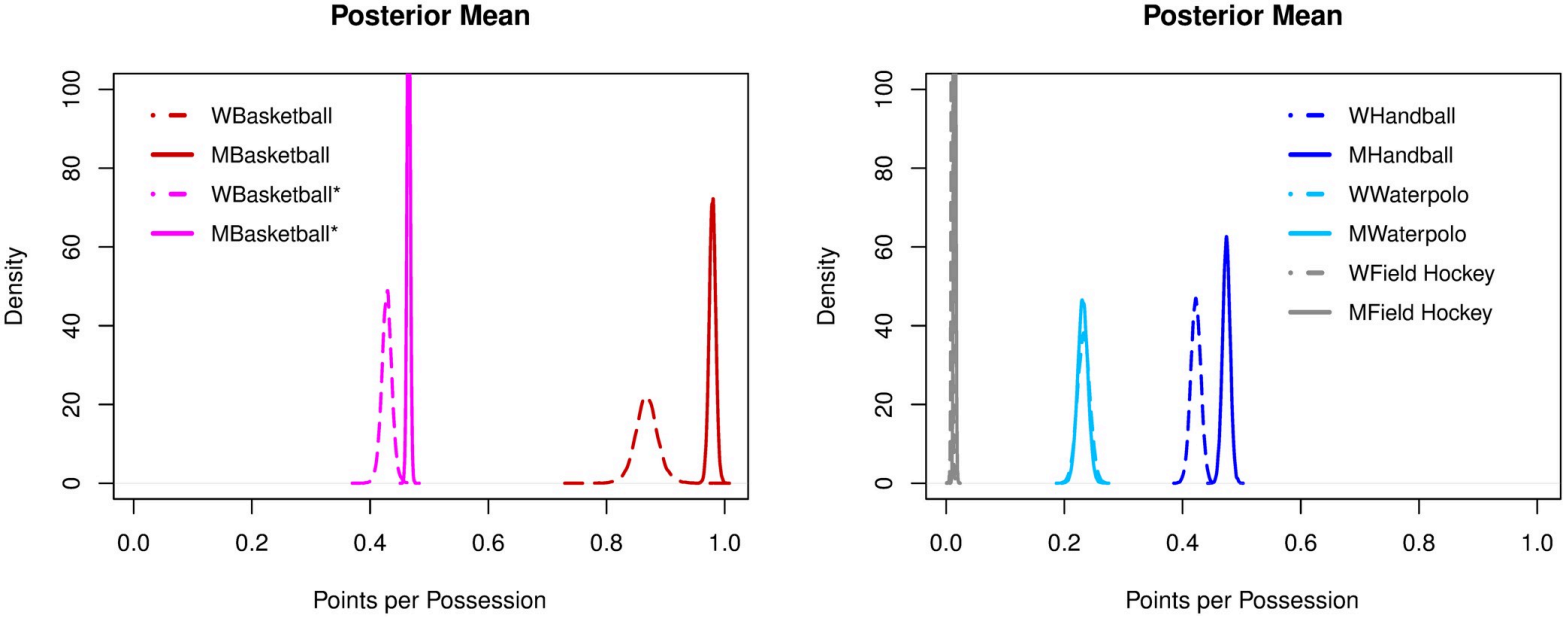
Posterior densities of the mean parameter of points per possession across genders.

Points per possession rates were significantly higher for men than for women in basketball (weighted and unweighted) and handball (p > 0.9). For water polo and field hockey, women and men had similar rates, see Fig 7.

### Efficiency: Correlations

Table 1 displays the correlations for every pair of scoring efficiency variables. Correlation of SHTpPOS-PTSpSHT varied from low (-0.17 to 0.43) for women’s basketball, men’s basketball, men’s handball, women’s field hockey, men’s field hockey and ice hockey to moderate (0.58 to 0.67) for women’s handball, women’s water polo, men’s water polo and football. Women’s water polo (0.67) and women’s handball (0.66) had significantly higher values than women’s basketball (0.32) and women’s field hockey (0.19).

**Table 1:** Point estimates for correlations of SHTpPOS-PTSpSHT, SHTpPOS-PTSpPOS, and PTSpSHT-PTSpPOS. Significant differences across sports, but within the same gender, are also noted.

	Bask		Hand		WaPo		FHck		IHck	Fball
	W	M	W	M	W	M	W	M	M	M
**1**	0.32	0.00	0.66	0.43	0.67	0.65	0.19	0.09	-0.17	0.58
	*h,p*	*h,p,o*	*b,f*	*b,f,k*	*b,f*	*b,f,k*	*h,p*	*h,p,o*	*h,p,o*	*b,f,k*
**2**	0.49	0.30	0.83	0.68	0.78	0.81	0.73	0.64	0.12	0.84
	*h,p*	*h,p,o*	*b*	*b,k*	*b*	*b,k*		*k*	*h,p,f,o*	*b,k*
**3**	0.86	0.89	0.94	0.93	0.93	0.94	0.55	0.49	0.91	0.82
		*f*	*f*	*f*	*f*	*f*	*h,p*	*b,h,p,k,o*	*f*	*f*

Row labels: 1—SHTpPOS-PTSpSHT; 2—SHTpPOS-PTSpPOS; 3—PTSpSHT-PTSpPOS. Initials of the sports (“b”: basketball; “h”: handball; “p”: water polo; “f”: field hockey; “k”: ice hockey; “o”: football) indicate significant differences (p > 0.9) for comparisons with sports of the same gender, that is, men’s basketball is compared to men’s handball.

Correlation of SHTpPOS-PTSpPOS were low for men’s basketball (0.30) and ice hockey (0.12), moderate for women’s basketball (0.49) and above 0.6 for all others (0.64—0.84). The highest correlations were in football (0.84), women’s handball (0.83) and men’s water polo (0.81), all significantly greater than both men’s and women’s basketball and ice hockey.

Correlation of PTSpSHT-PTSpPOS were high in all cases (≥ 0.82), except for both men’s (0.49) and women’s (0.55) field hockey.

## Discussion

This study compared the scoring efficiency among focused-target invasion team sports and between genders within the same sport. Sports presented either high (handball, basketball, water polo, ice hockey) or low (football, field hockey) probabilities of creating scoring opportunities combined with moderate (handball, basketball, water polo) or low (ice hockey, football, field hockey) probabilities of converting opportunties into points. High-creation sports presented greater asymmetry between the probabilities of creating an opportunity and converting a point in comparison to low-creation sports. Still, the lowest creation sport—football—had significantly greater correlation between creating and converting points than two of the highest creation ones—basketball and ice hockey. Between genders, men tended to outperform women more in creating opportunities than in converting opportunities into points.

Our results indicate that rates of ball possession vary significantly among sports and so do rates of creating scoring opportunities. We found the highest possession exchange rates for field hockey (men: 2.49; women: 3.10) and basketball (women: 2.26; men: 2.36), with significantly more ball possessions per minute than the other sports. In basketball, the shot clock undoubtedly contributes to higher possession rates [46]. Observed high exchanging rates in basketball does not seem to compromise performance in creating opportunities, given the high probability of taking a shot registered (Section 3.2.1—SHTpPOS probabilities). In field hockey, higher possession rates may be more related to frequently losing control of the ball to the opponent, since rates of creating opportunities are low. Findings for field hockey corroborate the importance atributed to the preparatory actions to a shot [47], with emphasis to passing and interception-related actions [48].

We also identified similar and moderate rates of possession exchanges in water polo (women: 1.31; men: 1.24), ice hockey (men: 1.31) and football (men: 1.50). Although water polo also has a shot clock, its highly intermittent dynamic [31, 49] and increasing effect of fatigue along the game [49] may contribute to slow the game pace. Moderate possession exchange rates seem to give appropriate support for high probabilities of creating opportunities both for water polo and ice hockey. In football, exchanging possession rates (men: 1.50) points to the challenge of dealing with ball conservation and pitch progression with the feet, given the low rate of creating shots [50]. Finally, handball’s lowest possession rates (women: 1.02; men: 0.96) appear to be well adjusted to support the high shot creating efficiency of this sport, leading to attempts of adjusting the game rules in order to increase its pace [14]. In summary, lower exchanging rates seem to be associated with better shot creation rate. Basketball is the exception and the position of the hoop may contribute to the patterns of high exchange and high creation opportunities.

Creating a shot opportunity (SHTpPOS) was identified as a frequent or a rare event, occurring more than six times or less than once every ten ball possessions, respectively, for high creation sports (p > 0.65—basketball, handball, ice hockey, water polo), or for low creation sports (p < 0.09—football, field hockey), corroborating previous evidences for some of these sports [10, 18, 50–52]

Handball and basketball (unweighted, as it is more appropriate for comparison with other sports) combine high shot creation rates with moderate rates of points conversion (PTSpSHT). Handball had the highest combined efficiency for creating (women: 0.76, men: 0.80) and converting shots (women: 0.55, men: 0.59). These results confirm previous suggestions of intermmediate convertion rates in this sport [53]. Handball was followed by basketball (unweighted), with shot creation and conversion rates of respectively 0.84-0.87 and 0.49-0.52. Consistently, basketball unweighted (women: 0.43, men: 0.46) and handball (women: 0.42; men: 0.47) had the highest scoring efficiency rates, with almost an opportunity per ball possession and nearly a score every two possessions.

Water polo and ice hockey combine high shot creation rates with low or extremely low conversion rates. Water polo had shot creation and conversion rates of, respectively, 0.66-0.67 and 0.34-0.33. Its scoring efficiency (women: 0.23; men: 0.23) numbers highlight the struggle for effectively converting a point in this game in comparison to taking a shot. It corroborates the positive effect of improving the quality of opportunities created to favor the conversion of points [54] and, specifically in water polo, it may imply getting closer to the goal in the central region of the pool [55]. Ice hockey evidenced the largest asymmetry between the creation of scoring opportunities (men: 0.73) and converting points (men:0.05), with an extremely low scoring efficiency (men: 0.03). Consistently with water polo (both high creation and moderate/low conversion sports) the quality of opportunity created, notably with better passing plays, is fundamental [56].

Football and field hockey had low rates both for creating opportunities and converting points. In football, the low rate of creating shots (men:0.09) highlights the challenge of finding an open line for a shot on goal [50, 57]. Nonetheless, if the shot is taken, the conversion rate (men:0.11) is slightly higher than the creation rate. The trend of a higher conversion rate than creation rate is also found in field hockey (SHTpPOS—men: 0.05, women: 0.04, PTSpSHT—men: 0.26, women: 0.21). It enforces the importance of finding opportunities in low creating sports. Both field hockey (women: 0.01; men: 0.01) and football (men: 0.01) had an expected rate of scoring a point (PTSpPOS) in one of every one hundred possessions, the lowest scoring efficiencies overall. Comparatively, while high creation sports should improve the quality of opportunities created, low creation sports should focus on increasing the frequency of opportunties.

Despite the heterogeneity in creating and converting opportunities, scoring efficiency in a ball possession (PTSpPOS) did not exceed 0.5 in any of these sports, when scoring weights were not considered. Thus, not scoring in a ball possession is generally the most likely outcome. This idea may seem to be counter-intuitive for high-creating sports, especially for basketball given its weighted scores.

In the comparison between genders, results indicated men were more efficient in creating opportunities (SHTpPOS) than women except in water polo. This may reflect game dynamics features, such as a higher game pace for the men [43] and wider shooting range, favoring higher creation efficiencies. The aquatic environment possibly presents some attenuation in this regard.

For conversion of points (PTSpSHT), men’s handball and men’s field hockey presented higher conversion efficiency than women. Water polo presented similar efficiency between genders, consistently to its results for SHTpPOS. In basketball, when points’ weights were not considered, basketball unweighted evidenced similar efficiency between genders, possibly related to the higher conversion rates of three point shots for men that increase the difference between genders when weighted points are considered.

In terms of scoring efficiency (PTSpPOS), men were more efficient in handball. In basketball, there was a decrement in the difference for the efficiency between men and women when points were unweighted. Gender differences were not observed for water polo and field hockey.

Despite previous evidences indicating men’s game as more efficient [34, 38], present findings prevent generalizations in this regard. Evidences suggest the differences of scoring efficiency between genders may vary according to the sport and scoring related variable—SHTpPOS or PTSpSHT. Although genders tend to be apart in their efficiencies to create opportunities, a possible consequence of their distinct game dynamics [34, 37, 38], men and women present closer performance in the efficiency to convert points.

For two sports with high shot creation rates—basketball and ice hockey—increasing the frequency of opportunities does not seem to be a key factor in offensive performance. These sports had the lowest correlations between both creating and converting (SHTpPOS-PTSpSHT—women’s basketball: 0.32, men’s basketball: 0.00, ice hockey: -0.17) and creating and scoring efficiency (SHTpPOS-PTSpPOS—women’s basketball: 0.49, men’s basketball: 0.30, ice hockey: 0.12). The weight of points favoring long distance field goals, in basketball, and the quick displacements associated to the low density of players in ice hockey favor taking shots frequently. On the other hand, the size of the hoop, in basketball, the similar size of goal and goalkeeper, in ice hockey, and other defensive constraints in both sports make shot conversion a key task.

Comparatively, handball and water polo, also high shot creation sports, had moderate to high rates for the same correlations (SHTpPOS-PTSpSHT—women’s handball: 0.66, men’s handball: 0.43, women’s water polo: 0.67, men’s water polo: 0.65; SHTpPOS-PTSpPOS—women’s handball: 0.83, men’s handball: 0.68, women’s water polo: 0.78, men’s water polo: 0.81). Results suggest that in these sports, difficulties in taking a shot, for instance, the physical barrier imposed by the defensive system, are counter-balanced by factors such as the short distance of several of shots. Thus, the ability to overcome the physical barrier of the defense appears to be fundamental for scoring.

In the low shot creation sports of field hockey and football, increasing shot frequency seem to positively impact scoring (SHTpPOS-PTSpPOS—women’s field hockey: 0.73, men’s field hockey: 0.64; men’s football: 0.84). Specifically, football had the highest relationship of shot creation with scoring efficiency (SHTpPOS-PTSpPOS), corroborating previous evidences in this regard [58].

Present results were obtained from end of matches’ box-score data, with low sensitivity to the in-game adaptive, non-linear properties [59, 60]. In this sense, the influence on teams’ scoring efficiency of specific circumstances (e.g. close versus unbalanced matches) and of context-related variables (e.g. game period, quality of opposition, match-status) were not addressed. Greater data resolution may enhance contextual information of scoring efficiency trends. For some of the sports, the assessment was limited by the lack of data (e.g. women’s football and women’s ice hockey). Additionally, we identified highly distinct collecting methods and protocols to summarize match information among sports. Despite the existance of particular traditions in each sport, more comparative studies may foment the debate about analytic procedures standardization.

## Conclusion

This research proposes an approach for comparing offensive performance across team sports taking in consideration variables related to the two complementary scoring challenges—creating opportunities and converting them into goal/point. Standadization of variables based on a per ball possession criteria was the alternative applied for performing comparisons. The study demonstrates women and men differences are few in terms of scoring efficiency, in most of the sports. The probabilites associated with creating opportunities for shots and scoring may enrich the comprehenssion about team sports’ contexts in which previous works identified transfers in decision-making skills [61, 62]. The enhanced knowledge about sports’ efficiencies in creating and converting shots may also provide some guidance to coaches into their frequent searches for strategic insights in the playing features of other team sports. Finally, results may support the design of practices by the coaches, particularly in youth sportive programs in which practitioners are exposed to tactical contents of diverse team sports. Future works should consider the influence of variables such as home-court, competition level and age groups on the scoring efficiencies in the different team sports.

## Supporting information

S1 File Box-score data. Structured dataset of box-score data from all sports in the sample.
(XLSX)
